# Optimization of Antibacterial Activity of *Perilla frutescens var. acuta* Leaf against *Staphylococcus aureus* Using Evolutionary Operation Factorial Design Technique

**DOI:** 10.3390/ijms12042395

**Published:** 2011-04-06

**Authors:** Dae-Hyun Kim, Young-Chan Kim, Ung-Kyu Choi

**Affiliations:** 1 School of Nano-Biotechnology & Chemical Engineering, Ulsan National Institute of Science and Technology, Ulsan 689–798, Korea; E-Mail: chunja2411@unist.ac.kr; 2 Korea Food Research Institute, Seongnam, Kyonggi, 463–746, Korea; E-Mail: yckim@kfri.re.kr

**Keywords:** *Perilla frutescens var. acuta* leaf, EVOP-factorial design technique, antibacterial activity, *Staphylococcus aureus* ATCC6538

## Abstract

This study was undertaken to optimize extraction using evolutionary operation-factorial (EVOP) design technique to elicit the antibacterial activity of *Perilla frutescens var. acuta* leaf against *Staphylococcus aureus* ATCC6538. Higher antibacterial activity was achieved at higher extraction temperature and over a longer extraction time. Antibacterial activity was not affected by differentiation of the ethanol concentration in the extraction solvent. The maximum antibacterial activity of ethanolic extract of *P. frutescens* leaf against *S. aureus* was obtained at 75 °C (R = −0.7904^**^) extraction temperature, 24 h (R = −0.7273^**^) extraction time, and 45% (R = −0.0635) ethanol concentration. The population of *S. aureus* was decreased from 7.535 log CFU/mL in the initial set to 4.865 log CFU/mL in the third set by EVOP factorial design technique, as well as to 2.600 log CFU/mL by extraction with ethyl acetate. Further, the ethyl acetate extract revealed the highest phenolic contents (111.3 ± 8.6 mg% of dry sample) as compared to the other extracts. Also, the scanning electronic microscopic study of the ethanolic extract of *P. frutescens* revealed potential detrimental effect on the morphology of *S. aureus*.

## 1. Introduction

With the increase of bacterial resistance to antibiotics, there is considerable interest in investigating the antimicrobial effects of plant extracts against a range of bacteria, to develop other classes of safe and natural antimicrobials useful for infection control or for the preservation of food [1]. *Staphyloccus aureus* is a versatile pathogen associated with a broad spectrum of infections in human beings and food industry. *S. aureus* is considered to be the second most common pathogen causing outbreaks of food poisoning followed by *Salmonella*. Official reports from the same state mentioned that in 1997, 88 laboratory diagnosed bacterial outbreaks were reported and *S. aureus* was primarily the agent representing 36.3% of the total outbreaks [2]. The presence of *S. aureus* has also been detected in various food samples [3,4] and its possible critical control point throughout processing was recently reviewed [5].

The main advantage of the evolutionary operation (EVOP) factorial design technique [6–8] is to develop a more effective approach for the optimization of n variable system which hybridize EVOP methodology [9,10] including response surface methodology (RSM) derived from orthogonal polynomial fitting techniques [11,12]. The identification and evaluation of natural products with optimized levels of antimicrobial activity for the control of infectious pathogens can be considered as an important international challenge to food and medicine industries.

*Perilla frutescens var. acuta* L., herb belongs to the family Labiatae. *P. frutescens* is an edible plant frequently used as one of the most popular garnishes and food colorants in China, Japan and Korea. The leaves of *P. frutescens var. acuta*, shown to be detoxicant, antitussive, antibiotic, and antipyretic [13,14], are also utilized as a folk medicine for treating intestinal disorders and allergies, particularly in traditional Chinese medical practice [15]. Although the biological activity of *P. frutescens var. acuta,* as well as its superior safety, is well documented,, there is no report available on the optimization of antibacterial activity of *P. frutescens var. acuta* leaf against *S. aureus* ATCC6538 using evolutionary operation-factorial design technique.

In the present study, therefore, the combined effects of extraction temperature, extraction time and the ethanol concentration on the antibacterial activity of *Perilla frutescens var. acuta* leaf were investigated using the EVOP-factorial design to maximize the antibacterial activity of *Perilla frutescens var. acuta* leaf against *S. aureus* ATCC6538.

## 2. Results

### 2.1. Optimization of Antibacterial Activity by EVOP-fs

The experimental conditions used in the first set of experiments, the corresponding antibacterial activities of cycle I and II, their differences and average values are presented in Table 1. The extraction temperature, extraction time and ethanol concentration of central point in first set (E_10_, E_20_) were 45 °C, 12 h and 45%, respectively [16]. The error limits, effects and the change in the mean effect were calculated and the results are shown in Table 2.

In the first set, the error limits for average, effects and changes in mean were 0.1739, 0.1235 and 0.1096, respectively. The change in mean effect was −0.2115. According to the decision-making procedure, after calculating the change in the mean effect and error limit, an examination was necessary to determine whether any change in the control (search level) experimental conditions would help to improve the objective function [10]. The optimum condition was achieved when the effect was smaller than the error limit, while the change in the mean effect was large. Moreover, because the dependent variables are the population of *S. aureus* ATCC6538, in which growth was suppressed by addition of *P. frutescens var. acuta* leaf extract, the optimum point was reached when the code of mean effect was negative.

The determination of the magnitude of the change in mean effect, which is negative and large, compared to the error limit, is a requirement in order to confirm the achievement of the optimum condition. Such a situation, where some of the effects are larger in comparison to the error limit, does not ensure that the condition at the search region (E_10_, E_20_) of first set is the actual optimum and a second set of experiments is called for.

In the second set (set II), the search level (E_10_, E_20_) was fixed at the best condition of Set I, at a level of E_14_ in which the number of *S. aureus* ATCC6538 was 6.050 log CFU/mL. The extraction temperature, extraction time and ethanol concentration of central point in second set (E_10_, E_20_) were 60 °C, 18 h and 60%, respectively. The experimental conditions and the results of Set II experiments are presented in Table 3, and the effects and error limits are shown in Table 4. In the second set, the error limits for average, effects and changes in mean was 0.1739, 0.1235 and 0.1096, respectively. The change in mean effect was 0.0870. Most effective antibacterial activity (4.890 log CFU/mL) was obtained at E_14_. The extraction temperature, extraction time and ethanol concentration of E_14_ point in second set were 75 °C, 24 h and 45%, respectively. In this case, not all of the effects were smaller than error limit, and the change in mean effect was smaller compared to the error limit even though it is positive. It has been reported that if all or any of the effects are larger than the error limits, the change in the experimental conditions may yield better results [9].

**Figure 1.**
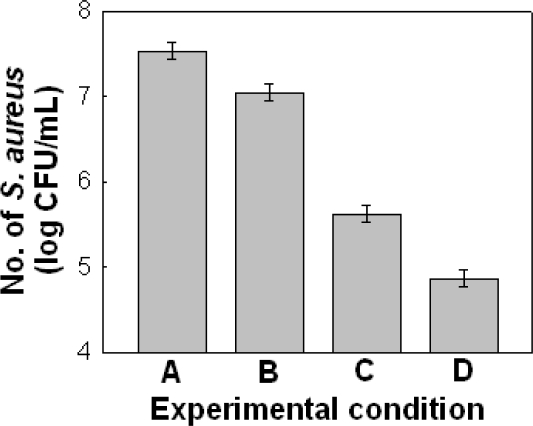
Comparison of antibacterial activity of *Perilla frutescens var. acuta* Leaf against *S. aureus* ATCC6538 at the central point of each set. **(A)** E_11_ of Set I (extraction temperature; 30 °C, extraction time; 6 hr, ethanol concentration; 30%); **(B)** central point of Set I (Extraction temperature; 45°C, extraction time; 12 hours, ethanol concentration; 45%); **(C)** central point of Set II (Extraction temperature; 60 °C, extraction time; 18 hours, ethanol concentration; 60%); **(D)** Central point of Set III (Extraction temperature; 75 °C, extraction time; 24 hours, ethanol concentration; 45%).

Under the above conditions, a third set of experiments was designed in which the best condition of Set II (E_14_) was selected as the search level (E_10_, E_20_) for Set III. The experimental conditions and the results of Set III are shown in Table 5, and the calculated effects and error limits are presented in Table 6. In the EVOP-factorial design, the effects remain smaller than the error limits while the changes in the mean effect remain larger and positive so as to reach the optimum level. Thus, in the experiments of third set (set III), we were able to arrive at the proper optimum condition, in which all effects were smaller than error limit and the changes in mean effect were large and positive. As shown in Figure 1, the population of *S. aureus* ATCC6538 decreased from 7.535 log CFU/mL in the initial set to 4.865 log CFU/ml in the third set.

**Figure 2.**
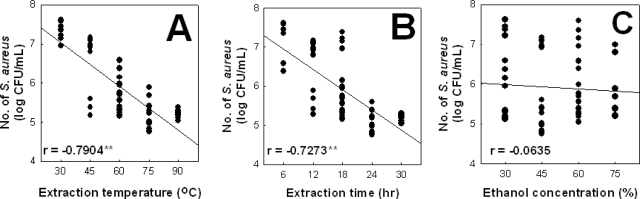
Main effects plot for responses against independent variables in EVOP (** p < 0.01). **(A)** extraction temperature; **(B)** extraction time; **(C)** ethanol concentration.

In this study, it was shown that higher antibacterial activity was achieved in a higher extraction temperature of 75 °C (R = −0.7904**) and in a longer extraction time of 24 h (R = −0.7273**). However, antibacterial activity of *P. frutescens var. acuta* leaf extract against *S. aureus* ATCC6538 was not affected by differentiation of ethanol concentration in the extraction solvent (R = 0.0635) as shown in Figure 2. Therefore, the maximum antibacterial activity of *P. frutescens var. acuta* leaf against *S. aureus* ATCC6538 determined by the EVOP-factorial technique was obtained at 75 °C extraction temperature, 24 h extraction time and 45% ethanol concentration.

**Figure 3.**
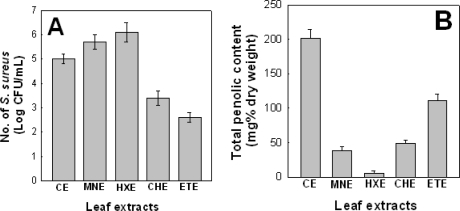
Antibacterial activity against *S. aureus* ATCC6538 and the amount of total phenolic content (mg% dry weight) of granic extracts derived from leaves of *Perilla frutescens var. acuta*. **(A)** Effect of *Perilla frutescens var. acuta* leaves on viability of *S. aureus* ATCC6538; **(B)** The amount of total phenolic content (mg% dry weight) of granic extracts derived from leaves of *Perilla frutescens var. acuta.*; MNE, methanol extract; HXE, hexane extract; CHE, chloroform extract; ETE, ethyl acetate extact.

*P. frutescens var. acuta* leaf extracted at optimum extraction condition (75 °C, 24 h, 45% ethanol concentration) was then extracted with 70% ethanol (MeOH), hexane, chloroform (CHCl_3_) and ethyl acetate (EtOAc) to identify *in vitro* antibacterial activities of *P. frutescens var. acuta* leaf against *S. aureus* ATCC6538. The effects of the *P. frutescens var. acuta* leaf extract on the growth of *S. aureus* ATCC6538 demonstrated the reduced viability. The ethyl acetate extracts exerted potential effect of antibacterial activity against *S. aureus* ATCC6538. The ethyl acetate extract exerted potential effect of antibacterial activity against *S. aureus* ATCC6538 followed by chloroform extract. More than 99% inhibition of tested pathogen was observed by the ethyl acetate extract (Figure 3A). Hexane and methanol extracts did not reveal significant effect of antibacterial activity against *S. aureus* ATCC6538.

Hexane extract and methanol extract did not reveal significant effect of antibacterial activity against *S. aureus* ATCC6538.

### 2.2. Total Phenolic Contents

The amount of total phenolic contents of the leaf extracts (n-hexane, chloroform, ethyl acetate and methanol) of *P. frutescens var. acuta* leaf was tested, and occurred in the range of 5.9–111.3 mg% dry sample (Figure 3B). The total phenolic contents of the leaf extracts of methanol, hexane, chloroform and ethyl acetate were noted to be 38.0 ± 6.7, 5.9 ± 3.2, 49.1 ± 4.3 and 111.3 ± 8.6 mg% of dry sample, respectively. These results showed that the total phenolic contents in ethyl acetate extract (111.3 ± 8.6 mg% of dry sample) were the highest as compared to the other extracts.

### 2.3. Scanning Electron Microscopy (SEM)

**Figure 4.**
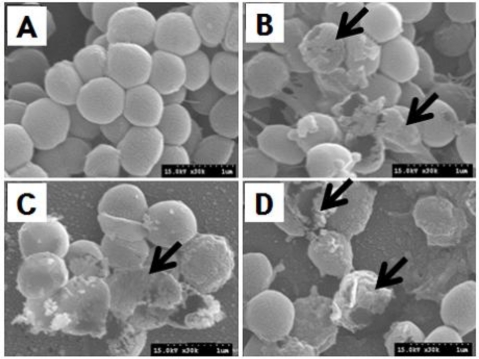
Scanning electron micrographs of *S. aureus* ATCC6538 at optimum concentration level of ethanolic leaf extract of *Perilla frutescens var. acuta* leaf. **(A)** Control; **(B)** disruption and lysis of membrane integrity; **(C)** wrinkled abnormalities and cleft formation; **(D)** abnormal breaking of cell.

Elaborative study of SEM was carried out to visualize the effects of the ethanolic extract of *P. frutescens var. acuta* leaf on the morphology of *S. aureus* ATCC6538 and demonstrated altered cell morphology as compared to control group (Figure 4). Control cells in the absence of the extract showed a regular, smooth surface (Figure 3A). In contrast, cells inoculated with the ethanolic extract of *P. frutescens var. acuta* leaf revealed severe detrimental effect on the morphology of cell membrane, showing disruption and lysis of the membrane integrity (Figure 4B). Exposure of *P. frutescens var. acuta* leaf extract to *S. aureus* ATCC6538 revealed large surface collapse and wrinkled abnormalities on the morphology of the cells along with some small clefts formation (Figure 4C and D) and these findings are in strong agreement with a previous report [17]. Besides, several researchers have reported the effects of various plant extracts on the morphology of pathogenic bacteria [17,18].

## 3. Experimental Section

### 3.1. Plant Material

The leaves of *P. frutescens var. acuta* were obtained from Yakrung market, Daegu, Republic of Korea, in June 2009. The specimen was lyophilized for 48–72 h after storage at −70 °C. Freeze dried samples were pulverized with a blender (HJM-7000, Hanil, Korea). Extra pure grade solvents were purchased from Daemyung Scientific Co., Daegu, Republic of Korea. Chemical reagents were obtained from Sigma Co. (St. Louis, MO, USA), unless otherwise stated.

### 3.2. Microorganism

*Staphylococcus aureus* ATCC6538 was used in the antibacterial assay. The strain was obtained from the Korea Food and Drug Administration (KFDA), Daegu, Republic of Korea. Active cultures for experimental use were prepared by transferring a loopful of cells from stock cultures to flasks and inoculated in Luria-Bertani (LB) broth medium at 37 °C for 24 h. Culture of the bacterial strain was maintained on LB agar medium at 4 °C.

### 3.3. Preparation of Extracts

To design an experiment for establishing proper extraction conditions by EVOP-factorial design technique, 20 g of sample was hydrolyzed and extracted in a reflux extraction apparatus by differentiating the extraction temperature (30, 45, 60, 75 and 90 °C), extraction time (6, 12, 18, 24 and 30 h) and ethanol concentration (30, 45, 60 and 75%) and then freeze dried. Samples were homogenized for 30 seconds and serially diluted with peptone water (Difco, USA), as needed for the determination of microbial populations.

Leaves of *P. frutescens var. acuta* extracted at optimum extraction condition (75 °C, 24 h, 45% ethanol concentration) were then extracted with 70% ethanol (MeOH), hexane, chloroform (CHCl_3_) and ethyl acetate (EtOAc) separately at room temperature and the solvents from the combined extracts were evaporated by vacuum rotary evaporator (EYELA N1000, Japan).

### 3.4. EVOP-Factorial Design Technique

The EVOP-factorial design technique was applied to select the optimum conditions of three extraction factors in different experiments [8]. First, the control or search level experimental conditions (E10, E20) were selected based on the results of early investigation on the effect of individual extraction condition on the antibacterial activity of the ethanolic extract of *P. frutescens var. acuta* leaf. In second phase, the new experimental conditions (Ebe) were selected with lower and higher levels of inducers compared to the search level (Eb0). Antibacterial activities of *P. frutescens var. acuta* leaf extract were estimated following the given assay procedure and recorded for cycle I and II. Differences in the antibacterial activities between cycle I and II, and average antibacterial activities were calculated to estimate the effects and error limits. The magnitudes of effects, error limits and change in mean effect were examined as per the decision making procedure to arrive at the optimum level. When the experimental results of the first set (set I) did not reach to the satisfactory level of optimum conditions, a second set (set II) of experiments was planned, selecting the best condition of the first set as the new search level for the second set. This procedure was repeated till the optimum condition was obtained.

### 3.5. Assay for Antibacterial Potential

To determine the antibacterial activity of *P. frutescens var. acuta* leaf extract, enumeration of viable counts on LB plates was monitored as followings: 1 mL of the resuspended culture was diluted into 9 mL buffer peptone water, thereby diluting 10-fold. In total, 0.1 mL sample of each treatment was diluted and spread on the surface of LB agar. The colonies were counted after 24 h of incubation at 37 °C.

### 3.6. Scanning Electron Microscopic (SEM) Analysis

To determine the efficacy of the ethanolic extract of *P. frutescens var. acuta* leaf on the morphology of *S. aureus* ATCC6538, SEM study was performed using optimum concentration level of the ethanolic extract of *P. frutescens var. acuta* leaf. Controls were prepared without extract. The method of SEM was modified from Kockro method to observe the morphological changes [18]. The bacterial samples were washed gently with 50 mM/L phosphate buffer solution (pH 7.2), fixed with 2.5%, 100 mL glutaraldehyde and 1%, 100 mL osmic acid solutions. The specimen was dehydrated using sequential exposure per ethanol concentrations ranging from 30–100%. The ethanol was replaced by tertiary butyl alcohol. After dehydration, the specimen was dried with CO_2_. Finally, the specimen was sputter-coated with gold in an ion coater for 2 min, followed by microscopic examinations (S-4300; Hitachi, Japan).

### 3.7. Determination of Total Phenolic Contents

The amount of total phenolics was determined with the Folin–Ciocalteu reagent [20]. First, a standard curve was plotted using gallic acid as a standard. Different concentrations of samples were prepared in 80% of methanol. 100 μL of sample was dissolved in 500 μL (1/10 dilution) of the Folin–Ciocalteu reagent and 1000 μL of distilled water. The solutions were mixed and incubated at room temperature for 1 min. After 1 min, 1500 μL of 20% sodium carbonate solution was added. The final mixture was shaken and then incubated for 2 h in the dark at room temperature. The absorbance of samples was measured at 760 nm and the results were expressed in mg of gallic acid/g (GAE) of dry weight of samples.

## 4. Discussion

This study evaluated the optimum condition for determining the antibacterial activity of *Perilla frutescens var. acuta* leaf against *Staphylococcus aureus* using evolutionary operation factorial design technique. The optimum antibacterial activity was obtained at 75 °C extraction temperature, 24 h extraction time and 45% ethanol concentration. From the above results, it can be concluded that the application of evolutionary operation factorial design technique could serve as a potential tool to determine the optimum extraction conditions to achieve the desired levels of antibacterial activity of natural products and their extracts for their potential utilization in food industry to control food-borne pathogenic bacteria. Further, the scanning electron microscopic study showed a potential detrimental effect of the *Perilla frutescens var. acuta* leaf extract on the morphology of *S. aureus* ATCC6538. These morphological features in bacterial cells might be due to the lysis of outer membrane and the transformation by weak peptidoglycan followed by the loss of cellular electron dense material on the surface of the treated cells, resulting in the release of inner cell materials [21,22].

Moreover, the results obtained in this study support the possible use of *Perilla frutescens var. acuta* leaf extracts in the food industry, where pathogenic bacteria causes severe destruction by hampering the quality of food and consumer demand. We hope that natural compounds such as *Perilla frutescens var. acuta* leaf extract might be a suitable candidate in food industry to serve as a natural preservative to control food-borne pathogens. Besides, phenolic compounds were found to be one of the most abundant classes of constituents in ethyl acetate extract of *Perilla frutescens var. acuta* leaves. This is due to the presence of high bioactive compounds in ethyl acetate extract as compared to other organic extracts [22,23].

In conclusion, the population of *S. aureus* ATCC6538 decreased from 7.535 log CFU/mL to 4.865 log CFU/mL using EVOP factorial design technique in which three extraction conditions (extraction temperature, extraction time, ethanol concentration) were differentiated. Further, the bacterial population continued to decrease to 2.600 log CFU/mL by extraction with ethyl acetate. This is the first report of the application of evolutionary operation factorial design technique to evaluate the antibacterial activity of *Perilla frutescens var. acuta* leaf extracts and should prove useful in finding optimal extraction conditions for antibacterial activities.

## Figures and Tables

**Table 1.** Experimental design for the three inducer system of Set I.

**Experimental conditions**	**E_10_**	**E_11_**	**E_12_**	**E_13_**	**E_14_**	**E_20_**	**E_21_**	**E_22_**	**E_23_**	**E_24_**
Temperature (°C)	45(0)	30(−)	30(−)	60(+)	60(+)	45(0)	60(+)	30(−)	60(+)	30(−)
Time (h)	12(0)	6(−)	18(+)	6(−)	18(+)	12(0)	18(+)	6(−)	6(−)	18(+)
Ethanol concentration (%)	45(0)	30(−)	60(+)	60(+)	30(−)	45(0)	60(+)	60(+)	30(−)	30(−)
Antibacterial activity (cycle I) (Log CFU/mL)	6.97	7.45	7.15	6.58	6.15	7.17	5.75	7.35	6.59	7.22
Antibacterial activity (cycle II) (Log CFU/mL)	7.12	7.62	6.96	6.39	5.95	6.92	5.99	7.59	6.37	7.38
Difference (cycle I − cycle II) (Log CFU/mL)	−0.15	−0.17	0.19	0.19	0.20	0.25	−0.24	−0.24	0.22	−0.16
Average activity (Log CFU/mL)	7.045 (a_10_)	7.535 (a_11_)	7.055 (a_12_)	6.485 (a_13_)	6.050 (a_14_)	7.045 (a_20_)	5.870 (a_21_)	7.470 (a_22_)	6.480 (a_23_)	7.300 (a_24_)

Note: Numbers in parentheses are the coded symbols of levels of the extraction conditions.

**Table 2.** Effect on three-variable system, magnitude and error limits of Set I.

**Effects of**		**Calculation of effects**	
Temperature		1/4(a_13_+a_14_+a_21_+a_23_−a_11_−a_12_−a_22_−a_24_)	0.1013
Time		1/4(a_12_+a_14_+a_21_+a_24_−a_11_−a_13_−a_22_−a_23_)	−0.4238
Ethanol concentration		1/4(a_12_+a_13_+a_21_+a_22_−a_11_−a_14_−a_23_−a_24_)	−0.1213
Temperature × Time		1/4(a_11_+a_14_+a_21_+a_22_−a_12_−a_13_−a_23_−a_24_)	−0.0988
Temperature × Ethanol concentration		1/4(a_11_+a_13_+a_21_+a_24_−a_12_−a_14_−a_22_−a_23_)	0.0338
Time × Ethanol concentration		1/4(a_11_+a_12_+a_21_+a_23_−a_13_−a_14_−a_22_−a_24_)	−0.0913
Temperature × Time × Ethanol concentration		1/4(a_21_+a_22_+a_23_+a_24_−a_11_−a_12_−a_13_−a_14_)	−0.5013
Change in mean effect		1/10(a_11_+a_12_+a_13_+a_14_+a_21_+a_22_+a_23_+a_24_−4a_10_−4a_20_)	−0.2115
Standard deviation (σ)		1/2(σ_1_+σ_2_)=1/2(R_1_ × f_k,n_ + R_2_ × f_k,n_)^(1)^	0.1230
Error limits :	For average	±1.414σ (±2σ/√n)	0.1739
	For effects	±1.004σ (±0.71 × 2σ/√n)	0.1235
	For change in mean	±0.891σ (±0.63 × 2σ/√n)	0.1096

R_1_: (largest difference—smallest difference) in Block 1; R_2_: (largest difference—smallest difference) in block 2. f_k,n_ = constant depending on number of replication (n) and number of experiments (k) per cycle = 0.3 for n = 2 and k = 5.

**Table 3.** Experimental design for the three inducer system of Set II.

**Experimental conditions**	**E_10_**	**E_11_**	**E_12_**	**E_13_**	**E_14_**	**E_20_**	**E_21_**	**E_22_**	**E_23_**	**E_24_**
Temperature (°C)	60(0)	45(−)	45(−)	75(+)	75(+)	60(0)	75(+)	45(−)	75(+)	45(−)
Time (h)	18(0)	12(−)	24(+)	12(−)	24(+)	18(0)	24(+)	12(−)	12(−)	24(+)
Ethanol concentration (%)	60(0)	45(−)	75(+)	75(+)	45(−)	60(0)	75(+)	75(+)	45(−)	45(−)
Antibacterial activity (cycle I) (Log CFU/mL)	5.70	6.95	5.18	5.89	4.78	5.76	5.23	6.80	5.42	5.60
Antibacterial activity (cycle II) (Log CFU/mL)	5.55	7.08	5.38	5.69	5.00	5.57	5.40	6.99	5.28	5.39
Difference (cycle I − cycle II) (Log CFU/mL)	0.15	−0.13	−0.20	0.20	0.22	0.19	−0.17	−0.19	0.19	0.21
Average activity (Log CFU/mL)	5.625 (a_10_)	7.015 (a_11_)	5.280 (a_12_)	5.790 (a_13_)	4.890 (a_14_)	5.665 (a_20_)	5.315 (a_21_)	6.895 (a_22_)	5.350 (a_23_)	5.495 (a_24_)

Note: Numbers in parentheses are the coded symbols of levels of the extraction conditions.

**Table 4.** Effects on three-variable system, magnitude and error limits of Set II.

**Effects of**		**Calculation of effects**
Temperature		−0.8350
Time		−1.0175
Ethanol concentration		0.1325
Temperature × Time		0.5500
Temperature × EC		0.3000
Time × Ethanol concentration		−0.0275
Temperature × Time × EC		−0.1638
Change in mean effect		0.0870
Standard deviation (σ)		0.1230
Error limits :	For average	0.1739
	For effects	0.1235
	For change in mean	0.1096

R_1_: (largest difference—smallest difference) in Block 1; R_2_: (largest difference—smallest difference) in block 2. f_k,n_ = constant depending on number of replication (n) and number of experiments (k) per cycle = 0.3 for n = 2 and k = 5.

**Table 5.** Experimental design for the three inducer system of Set III.

**Experimental conditions**	**E_10_**	**E_11_**	**E_12_**	**E_13_**	**E_14_**	**E_20_**	**E_21_**	**E_22_**	**E_23_**	**E_24_**
Temperature (°C)	75(0)	60(−)	60(−)	90(+)	90(+)	75(0)	90(+)	60(−)	90(+)	60(−)
Time (h)	24(0)	18(−)	30(+)	18(−)	30(+)	24(0)	30(+)	18(−)	18(−)	30(+)
Ethanol concentration (%)	45(0)	30(−)	60(+)	60(+)	30(−)	45(0)	60(+)	60(+)	30(−)	30(−)
Antibacterial activity (cycle I) (Log CFU/mL)	4.83	5.15	5.18	5.38	5.11	4.76	5.26	5.25	5.31	5.30
Antibacterial activity (cycle II) (Log CFU/mL)	5.02	5.35	5.30	5.19	5.21	4.97	5.05	5.40	5.18	5.20
Difference (cycle I − cycle II) (Log CFU/mL)	−0.19	−0.20	−0.12	0.19	−0.10	−0.21	0.21	−0.15	0.13	0.10
Average activity (Log CFU/mL)	4.925 (a_10_)	5.250 (a_11_)	5.240 (a_12_)	5.285 (a_13_)	5.160 (a_14_)	4.865 (a_20_)	5.155 (a_21_)	5.325 (a_22_)	5.245 (a_23_)	5.250 (a_24_)

Note: Numbers in parentheses are the coded symbols of levels of the extraction conditions.

**Table 6.** Effects on three-variable system, magnitude and error limits of Set III.

**Effects of**		**Calculation of effects**
Temperature		−0.0550
Time		−0.0750
Ethanol concentration		0.0236
Temperature × Time		−0.0325
Temperature × EC		−0.0075
Time × Ethanol concentration		−0.0325
Temperature × Time × EC		0.0100
Change in mean effect		0.2750
Standard deviation (σ)		0.1215
Error limits :	For average	0.1718
	For effects	0.1299
	For change in mean	0.1083

R_1_: (largest difference—smallest difference) in Block 1; R_2_: (largest difference—smallest difference) in block 2. f_k,n_ = constant depending on number of replication (n) and number of experiments (k) per cycle = 0.3 for n = 2 and k = 5.
